# Gender Differences Are Encoded Differently in the Structure and Function of the Human Brain Revealed by Multimodal MRI

**DOI:** 10.3389/fnhum.2020.00244

**Published:** 2020-07-21

**Authors:** Xi Zhang, Meng Liang, Wen Qin, Baikun Wan, Chunshui Yu, Dong Ming

**Affiliations:** ^1^Department of Biomedical Engineering, College of Precision Instruments and Optoelectronics Engineering, Tianjin University, Tianjin, China; ^2^School of Medical Imaging, Tianjin Medical University, Tianjin, China; ^3^Tianjin Key Laboratory of Functional Imaging, Tianjin Medical University, Tianjin, China; ^4^Department of Radiology, Tianjin Medical University General Hospital, Tianjin, China; ^5^Academy of Medical Engineering and Translational Medicine, Tianjin University, Tianjin, China

**Keywords:** gender difference, gray matter volume, regional homogeneity, functional connectivity, multivariate pattern analysis, support vector machine

## Abstract

Despite widely reported gender differences in both brain structure and brain function, very few studies have examined the relationship between the structural differences and the functional differences between genders. Here, different imaging measures including both structural [i.e., gray matter volume (GMV)] and functional [i.e., regional homogeneity (ReHo) and functional connectivity (FC)] measures were employed to detect the gender differences in the human brain based on univariate and multivariate approaches with a sample of 290 healthy adults (155 females). The univariate analyses revealed that gender differences were detected in both structural (i.e., GMV) and functional (ReHo or FC) imaging measures, mainly manifested as greater values in females than in males in regions of the frontal, parietal, occipital lobes and cerebellum. Importantly, there was little overlap between the differences detected in GMV and those detected in ReHo and FC, and their differences between genders were not correlated with each other. The multivariate pattern analyses revealed that each of these measures had discriminative power to successfully distinguish between genders (classification accuracy: 94.3%, 90.73%, and 83.89% for GMV, ReHo, and FC, respectively) and their combination further improved the classification performance (96.6%). Our results suggest that gender differences are encoded in both brain structure and brain function, but in different manners. The finding of different and complementary information contained in structural and functional differences between genders highlights the complex relationship between brain structure and function, which may underlie the complex nature of gender differences in behavior.

## Introduction

There is a wide range of evidence for gender differences in behavioral profiles as well as in brain structure and function (Sacher et al., 2013; Ruigrok et al., 2014; Gur and Gur, 2017). Behaviorally, males are shown to perform superiorly in some domains including motor and visuospatial processing, whereas females have an advantage in terms of verbal skills and emotional memory. There is an increasing interest in studying the brain mechanisms underlying these behavioral differences between genders. For example, there is evidence showing that larger gray matter volume (GMV) in occipital lobe was correlated with better visual function in males and larger hippocampal gyrus was correlated with better memory performance in females (Giedd et al., 2012). Given that gender differences in patients’ prevalence and symptoms are commonly seen in many neuropsychiatric disorders, a better understanding of the gender differences in brain structure and function could also provide insights into the neurophysiological mechanisms of clinical symptoms and help improve the outcome of clinical interventions.

Previous studies have utilized a variety of brain imaging measures derived from different magnetic resonance imaging (MRI) modalities and different analytical techniques including conventional univariate methods and recently developed multivariate methods to examine gender differences in brain structure and function. Univariate methods examine the differences between genders in a voxel-wise or region-wise manner, i.e., performing statistical comparisons between genders for every voxel or region in the brain. For instance, some studies on gender differences in GMV using voxel-based morphometry (VBM) reported that females had a larger amount of gray matter (GM) in the frontal and parietal cortices than males (Sacher et al., 2013; Ruigrok et al., 2014). In contrast, multivariate methods, most typically, the multivariate pattern analysis (MVPA), examines the difference in spatial patterns across multiple voxels or regions. As MVPA considers multiple voxels/regions at once, it can extract more information from the data and thus is more sensitive in detecting differences between experimental conditions or groups than univariate methods (Norman et al., 2006; Pereira et al., 2009; Liu et al., 2015). MVPA is a machine learning technique and can be used for classification between different groups (e.g., males vs. females) or prediction of behavioral performance. Indeed, both structural and functional brain imaging measures have been used to distinguish participants between genders in previous studies. For example, a classification between genders based on brain structural connectome resulted in an overall classification accuracy (CA) of 79% (Tunc et al., 2016). Moreover, patterns of functional connectivity (FC) have also been shown to be distinguishable between genders, yielding a 71% CA which is higher than that obtained from participants’ cognitive profiles (63%; Satterthwaite et al., 2015). All these findings show that males and females differ in both brain structure and function.

However, it remains unclear whether gender differences in brain structure and brain function show similar patterns, that is, whether females and males differ structurally and functionally in similar brain regions. Different measures obtained from multimodal brain imaging data likely contain not only shared but also complementary information about gender differences. Addressing this question requires a comparison of gender differences detected by different imaging measures obtained from multimodal MR data in the same group of participants. However, most previous studies only focused on a single measure of the brain, either structural or functional, in any particular study. The complex relationship between structural and functional imaging metrics has been identified in normal population (Yang et al., 2016) and was found to be altered in certain clinical populations such as patients with generalized tonic-clonic seizures (Liao, 2016). Regarding gender differences, a recent study with a large sample size (*n* = 5,216) examined gender differences in a variety of MR metrics including structural, diffusion and functional measures in the adult brain and confirmed that females and males differ in both brain structure and function using the same group of subjects (Ritchie et al., 2018). However, the similarities and differences between the gender differences in brain structure and those in brain function have not been formally investigated. Furthermore, the samples used in this recent study were mainly middle-aged or old people (ranging from 44 to 77 years), and thus their results might not apply to young adults given that gender differences in brain structure and function may be age-related (Kawachi et al., 2002; Sowell et al., 2007; Zuo et al., 2010; Gennatas et al., 2017).

In the present study, we performed multimodal MR imaging of 135 males and 155 females and extracted voxel-based GMV from structural MRI and regional homogeneity (ReHo) and FC from resting-state functional MRI (fMRI) of each participant. We then identified gender differences in these different imaging measures using both univariate and multivariate methods. We aimed to characterize the shared and different features of gender differences reflected by these different imaging measures and to test whether combining information provided by different measures could improve the accuracy of gender classification.

## Materials and Methods

### Participants

Three hundred and twenty-four healthy right-handed volunteers, confirmed for the absence of any neurological or psychiatric disorders, participated in this study. The handedness of each participant was assessed using the Chinese handedness questionnaire published by Li (1983). No brain lesions were observed during brain MRI data acquisition. Eight subjects were further excluded due to poor image quality, seven subjects were excluded due to excessive head motion (the maximal displacement ≥2 mm in x, y, or z direction, or maximal rotation ≥2.0° around any of the three axes) and nineteen subjects were excluded due to missing personality scale data. The remaining 290 subjects include 135 men (age range: 18–29 years; mean ± SD = 22.17 ± 2.49) and 155 women (age range: 18–29 years; mean ± SD = 23.26 ± 2.25). This study was approved by the Medical Research Ethics Committee of Tianjin Medical University General Hospital and all participants provided informed consent before the experiment.

### MRI Data Acquisition

Each subject underwent a resting-state fMRI scan followed by a T1-weighted anatomical scan (3.0 T, General Electric, Milwaukee, WI, USA). Resting-state fMRI data covering the entire brain were acquired using a single-shot, echo-planar imaging (EPI) sequence with the following parameters: repetition time/echo time (TR/ TE) = 2,000/30 ms, flip angle (FA) = 90°, matrix = 64 × 64, the field of view (FOV) = 240 × 240 mm^2^, 40 axial slices, slice thickness = 4 mm, no gap. Each functional run contained 180 volumes. During the fMRI scanning, all subjects were informed to keep their eyes closed, stay awake, and not to think of anything in particular. To mitigate motion and noise, participants’ heads were made stable in the head coil through a foam pad and earplug in each ear. Finally, a high-resolution T1-weighted brain volume (BRAVO) 3D MRI sequence with 176 contiguous sagittal slices was performed with the following parameters: TR/TE = 8.1/3.1 ms, inversion time = 450 ms, FA = 13°, matrix = 256 × 256, FOV = 256 × 256 mm^2^, slice thickness = 1 mm without a gap. This structural brain image was used to extract GMV.

The GMV maps were constructed for each subject using VBM analysis. All data pre-processing was performed using the VBM8 toolbox^1^ combined with the SPM12 software^2^. During the segmentation, an adaptive Maximum A Posterior technique (Rajapakse et al., 1997) and a Partial Volume Estimation (Tohka et al., 2004) were applied to estimate the fraction of each pure tissue type present in every voxel. Following the segmentation of GM, white matter (WM) and cerebrospinal fluid (CSF), the individual GM concentration maps were normalized into GM template in the Montreal Neurological Institute (MNI) space using the diffeomorphic anatomical registration through the exponentiated Lie algebra (DARTEL) algorithm (Ashburner, 2007) and re-sliced to a voxel size of 1.5 × 1.5 × 1.5 mm^3^. In the modulation process, the GMV maps of each subject were obtained by multiplying the individual’s GM concentration map by the nonlinear Jacobian determinants derived from the spatial normalization while removing the confounding effect of variance in individual brain sizes. Finally, the GMV maps were smoothed with a 4-mm full width at half maximum (FWHM) Gaussian kernel for increasing the signal-to-noise ratio. Finally, the normalized and smoothed GMV maps were resampled to 3 × 3 × 3 mm^3^ voxel size to match with the voxel size of functional data.

### Regional Homogeneity and Functional Connectivity Obtained From Functional MRI Data

#### Preprocessing

fMRI data were preprocessed by the software package Data Processing Assistant for Resting-State fMRI (DPARSFA^3^). The first 10 volumes were discarded to ensure signal stabilization and the remaining 170 volumes were corrected for the acquisition time delay between different slices. Then all volumes were aligned with each other in each subject to correct head motion. All aligned volumes were spatially normalized to the standard EPI template and resampled at 3 × 3 × 3 mm^3^ voxels. The normalized data were smoothed with a 4-mm FWHM Gaussian kernel. Next, the band-pass filtering (between 0.01 and 0.08 Hz) was applied to remove the effect of low-frequency drift and high-frequency noises. Finally, several factors including linear drift, the six parameters of head motion, the average blood oxygen level-dependent (BOLD) signals of the whole brain and the average signals of the ventricular and WM regions were regressed out as covariates of no interest from the BOLD time series of each voxel (Fox et al., 2009; Liu et al., 2015). Only the voxels within a GM mask (available in the software package DPARSFA, including 67,541 voxels) were entered into the subsequent univariate comparisons and classification analyses between genders.

#### Regional Homogeneity

The regional homogeneity (ReHo) is a measure of local synchronization of intrinsic fMRI signals by calculating Kendall’s coefficient of concordance (KCC) between the time series of a given voxel and those of its 26 neighboring voxels (Zang et al., 2004). This measure has been widely used to reveal information about spontaneous neural activity within a local region during rest. A ReHo map was obtained for each participant and then standardized (dividing the ReHo value of each voxel by the mean ReHo value of all voxels within the brain) and smoothed (with a Gaussian kernel of 4-mm FWHM) using the software package DPARSFA. Only the voxels within the GM mask were entered into the subsequent univariate comparisons and classification analyses between genders.

#### Functional Connectivity

The automated anatomical labeling (AAL) atlas was first used to partition the whole brain into 116 regions, including 90 regions in the cerebrum and 26 regions in the cerebellum. The mean time series were then obtained for each region in each subject by averaging the time series of all GM voxels within the given region. GM voxels were defined by the GM mask provided in the software package DPARSFA. The FC between any two regions was calculated as the Pearson’s correlation coefficient between their time series of fMRI signals, resulting in (116 × 115)/2 = 6670 functional connections of the whole brain. Then a Fisher’s r-to-z transformation was performed to transform the correlation coefficient to *Z* values to improve normality.

### Univariate Comparisons of Different Imaging Measures Between Genders

To explore the gender differences in brain structure and function, voxel-wise comparisons between genders were performed using two-sample *t*-tests for the GMV maps and ReHo maps separately within the GM mask. The final statistical results were corrected using the voxel-level family-wise error (FWE) method (*P* < 0.05, corrected). Similarly, for each of the FCs, the FC values of the male group and the female group were compared using a two-sample *t*-test. This comparison was repeated for every FC. The FCs with *P* < 7.50 × 10^−6^ (i.e., 0.05/6,670, Bonferroni corrected) were considered to be significantly different between genders. In all comparisons, confounding factors such as age and education years were included as covariates. For FC, we also evaluated the gender differences in the weighted degree of each brain region associated with the FCs showing significant gender differences. Here, the weight of each FC was its *T*-value obtained from the two-sample *t*-test. As *T*-values can be positive or negative, a positive weighted degree and a negative weighted degree were calculated separately for each brain region using the following procedure: for a given brain region, the positive weighted degree was calculated as the sum of the positive *T*-values of all FCs showing significant gender differences associated with this region; similarly, the negative weighted degree was calculated as the sum of the negative *T*-values of all FCs showing significant gender differences associated with this region. Note that, only the FCs identified to be significantly different between genders were used in the calculation of the weighted degree. Here, positive weights indicate greater strength in females, while negative weight indicates greater strength in males.

To formally analyze the similarities and differences between the gender differences in the structural measure (i.e., GMV) and the gender differences in the functional measure (i.e., ReHo), we examined the overlap between the thresholded GMV difference map and the thresholded ReHo difference map. To provide a more quantitative measure of the overlap, we calculated the percentage of the overlapping area in two ways: (1) the percentage of the overlapping voxels (i.e., the voxels showing significant differences in both GMV and ReHo between genders) over all voxels showing significant differences in GMV; and (2) the percentage of the overlapping voxels over all voxels showing significant differences in ReHo. As the thresholded map used in the above overlapping analyses were corrected for multiple comparisons to control the type I (i.e., false-positive) error, the false-negative rate might be high (i.e., there might be voxels which showed a trend of gender differences but did not survive the corrected threshold). For example, for the voxels showing significant differences in GMV but not in ReHo, there might be a high probability that there were also differences in ReHo in these voxels but the differences were not strong enough to survive the corrected threshold. Therefore, to provide a more thorough quantification of the overlapping area between the GMV difference and the ReHo difference, we further identified: (1) the voxels showing a trend of differences in ReHo (*P* < 0.001, uncorrected) in the properly thresholded GMV difference map (i.e., *P* < 0.05, corrected); and (2) the voxels showing a trend of differences in GMV (*P* < 0.001, uncorrected) in the properly thresholded ReHo difference map (i.e., *P* < 0.05, corrected). Besides, we also quantified the similarity between the GMV difference map and the ReHo difference map using Pearson’s correlation coefficient by performing a spatial correlation analysis between the two maps within the GM mask. As it was difficult to quantify the similarities and differences between gender differences in GMV/ReHo (measured for each voxel) and gender differences in FC (measured between regions), the above overlapping and spatial correlation analyses were only performed between GMV and ReHo.

### Gender Classification Using Brain Imaging Measures

The MVPA for gender classification was performed using an SVM classifier implemented in the LIBSVM toolbox (Chang and Lin, 2011) running in MATLAB platform (The Math Works, Inc., Natick, MA, USA). SVM with the linear kernel is widely used in MVPA of brain imaging data as it works well when the dimensionality of the feature space is much larger than the sample size (Pereira et al., 2009).

Gender classifications were performed using brain imaging metrics including GMV (67,541 features), ReHo (67,541 features), and FC (6,670 features) separately and also using the combination of the three features (141,752 features), thus leading to a total of four classification analyses. Before these classification analyses, the effects of age and education years were removed from each feature for each brain imaging metrics using multiple linear regression. For the classification using combined brain imaging metrics, each of the three metrics (i.e., GMV, ReHo, and FC) was first normalized to zero mean and unit variance using the mean and the standard deviation across all features of the same metric (i.e., Z transformation) for each subject. After normalization, the three feature vectors of each participant were concatenated into a single vector and then entered into the classification analysis.

For each classification analysis, the CA was determined using 10-fold cross-validation (CV) procedure as follows. The data were divided into 10 folds: each of the first nine folds contained 13 males and 15 females and the last fold contained all remaining subjects (i.e., 18 males and 20 females). During the CV process, nine folds were used to train the classifier, leaving the remaining one fold out for testing the trained classifier. This procedure was repeated 10 times so that each of the 10 folds was used as a test dataset once. One CA was obtained for each CV step, and the CAs obtained from all folds were averaged to obtain an overall CA of the entire dataset. Similarly, the overall sensitivity and specificity of all folds were also obtained. Here, sensitivity was defined as the percentage of male participants that were correctly classified as male, and specificity was defined as the percentage of female participants that were correctly classified as female. Furthermore, the receiver operating characteristic (ROC) curve and the corresponding area under the curve (AUC) were evaluated for each classifier to delineate the discriminative power.

Note that a feature selection procedure was embedded in the above CV procedure to identify the most discriminative feature sets that produce the highest CA. Specifically, for each CV step, all features were ranked in descending order according to their absolute weights obtained from the training step. For the classification using GMV (or ReHo), the number of the selected features with the highest rankings increased from 2,000 to the whole set of features (i.e., 67,541) in steps of 2,000, resulting in 34 selected feature sets. For the classification using FCs, the number of the selected features with the highest rankings increased from 100 to the whole set of features (i.e., 6,670) in steps of 100, resulting in 67 selected feature sets. For the classification using combined features, the number of the selected features with the highest rankings increased from 2,000 to the whole set of features (i.e., 141,752) in steps of 2,000, resulting in 71 selected feature sets. For each feature selection step, a CA was calculated for each CV step (i.e., fold) and then an average CA across all folds was calculated. The statistical significance of the CAs was determined by nonparametric permutation tests (*n* = 2,000). In brief, the same MVPA procedure was performed except that: (1) the labels of the training samples in each CV step were shuffled at random, and then the SVM model was trained using the randomly labeled training data and tested using the test set, with the same feature selection procedure; (2) this procedure was performed for every CV step, resulting in an average accuracy obtained at chance level; and (3) the whole permutation procedure was repeated 2,000 times, resulting in 2,000 average accuracies which were used to build a null distribution of chance-level accuracies. Therefore, the *P*-value of the actual CA obtained from the true labels was calculated by comparing it with the corresponding null distribution of chance level accuracies, that is, the *P*-value was the percentage of the chance-level accuracies that were greater than or equal to the actual accuracy. If none out of 2,000 permutations reached the actual accuracy, the *p*-value was labeled as *p* < 0.0005 (i.e., 1/2,000). Given that a different percentage in each feature selection was used, the procedure should be considered as multiple independent MVPA analyses and thus the above *P* values acquired from the permutation tests were further corrected for multiple comparisons with Bonferroni correction method (e.g., *P* < 0.05/71 = 0.0007 was considered to be statistically significant for the classification using combined features).

## Results

### Demographics

Significant differences in age (male: Mean ± SD = 22.17 ± 2.49; female: 23.26 ± 2.25; *T* = 2.5, *P* = 0.01; two-sample *t*-test) and education years (male: Mean ± SD = 15.13 ± 2.33; female: 15.86 ± 2.61; *T* = 1.9, *P* = 0.02; two-sample *t*-test) between the two groups were observed, and thus the effects of age and education years were removed as covariates in both univariate and multivariate comparisons between genders.

### Gender Differences in GMV, ReHo, and FC Identified by Univariate Analyses

**Figure 1:**
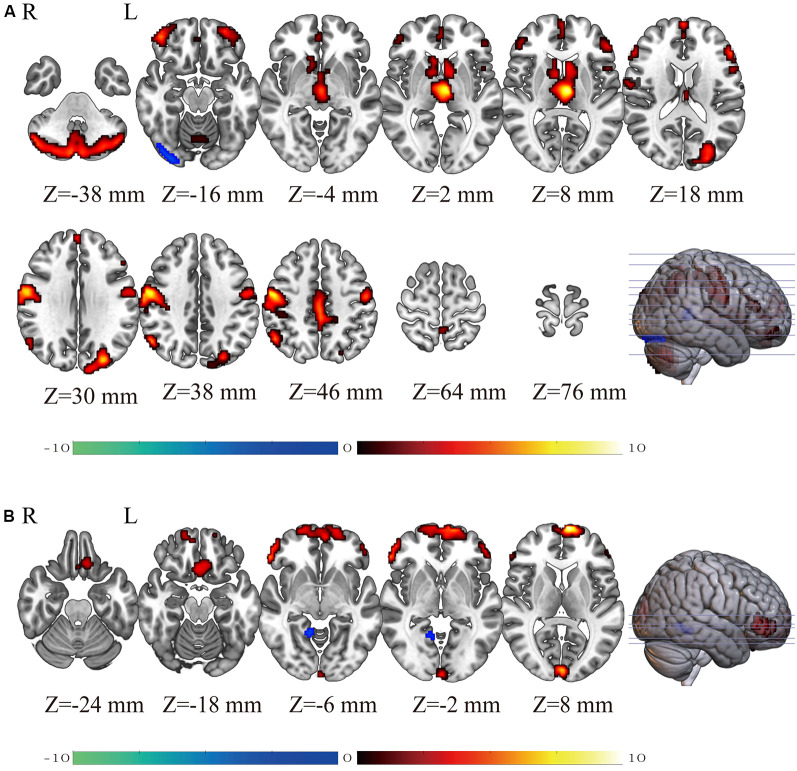
The brain maps showing gender differences (Female-Male) in gray matter volume (GMV; panel **A**) and regional homogeneity (ReHo; panel **B**) detected using two-sample *t*-tests. The *T*-values of the voxels showing significant differences are indicated in color. Warm colors (red-yellow) indicate higher GMV (panel **A**) or higher regional homogeneity (ReHo; panel **B**) in females than in males and cold colors (blue-green) indicate higher GMV (panel **A**) or higher ReHo (panel **B**) in males than in females.

Figure 1A and Table 1 show the gender differences in GMV. Females had greater GMV in several areas including the thalamus, postcentral gyrus, triangular part of inferior frontal gyrus, orbital part of middle frontal gyrus and medial superior frontal gyrus in both hemispheres, middle occipital gyrus and middle cingulate gyrus in the left hemisphere, and the inferior parietal lobule and caudate in the right hemisphere, and bilateral cerebellum. Males had greater GMV than females only in the right inferior occipital gyrus.

**Table 1:** Brain areas showing gender differences in gray matter volume detected by univariate two-sample *t*-tests (*P* < 0.05, FWE corrected).

Anatomical area	Brodmann area	Side	MNI coordinates	T (at peak voxel)
***Males > Females***			
Inferior Occipital Gyrus	18	R	(30, −90, −21)	−7.02
***Females > Males***				
Thalamus	-	L, R	(−6, −12, 6)	9.45
Postcentral gyrus	3	L	(−54, −6, 36)	5.76
Postcentral gyrus	3	R	(54, −9, 45)	8.35
Middle frontal gyrus, orbital part	11	R	(42, 51, −15)	7.87
Superior frontal gyrus, medial	10	L, R	(0, 63, 18)	6.78
Middle occipital gyrus	18	L	(−27, −84, 30)	7.58
Middle cingulate	31	L	(0, −36, 48)	6.73
Cerebelum_2	-	L	(−27, −78, −42)	6.99
Cerebelum_2	-	R	(48, −69, −42)	7.0
Middle frontal gyrus, orbital part	11	L	(−33, 54, −15)	6.73
Inferior frontal gyrus, triangular part	46	L	(−51, 39, 9)	5.84
Inferior frontal gyrus, triangular part	46	R	(45, 45, 6)	4.89
Inferior Parietal Lobule	40	R	(51, −57, 42)	5.15
Caudate	-	R	(6, 6, 9)	5.52

Figure 1B and Table 2 show the gender differences in ReHo. Females had greater ReHo in the medial superior frontal gyrus in both hemispheres, the triangular part of inferior frontal gyrus, rectus, and calcarine in the left hemisphere and the orbital part of inferior frontal gyrus in the right hemispheres. Males showed greater ReHo than females only in the right lingual gyrus.

**Table 2:** Brain areas showing sex differences in ReHo detected by univariate two-sample *t*-tests (*P* < 0.05, FWE corrected).

Anatomical area	Brodmann area	Side	MNI coordinates	T
***Males > Females***				
Lingual gyrus	18	R	(12, −51, −6)	−5.41
***Females > Males***				
Calcarine	18	L	(0, −93, 12)	8.69
Rectus	10	L	(0, 18, −15)	6.13
Superior frontal gyrus, medial	11	L, R	(−9, 69, 6)	10.57
Inferior frontal gyrus, triangular part	47	L	(−48, 48, 0)	5.94
Inferior frontal gyrus, orbital part	47	R	(57, 36, −6)	7.88

**Figure 2:**
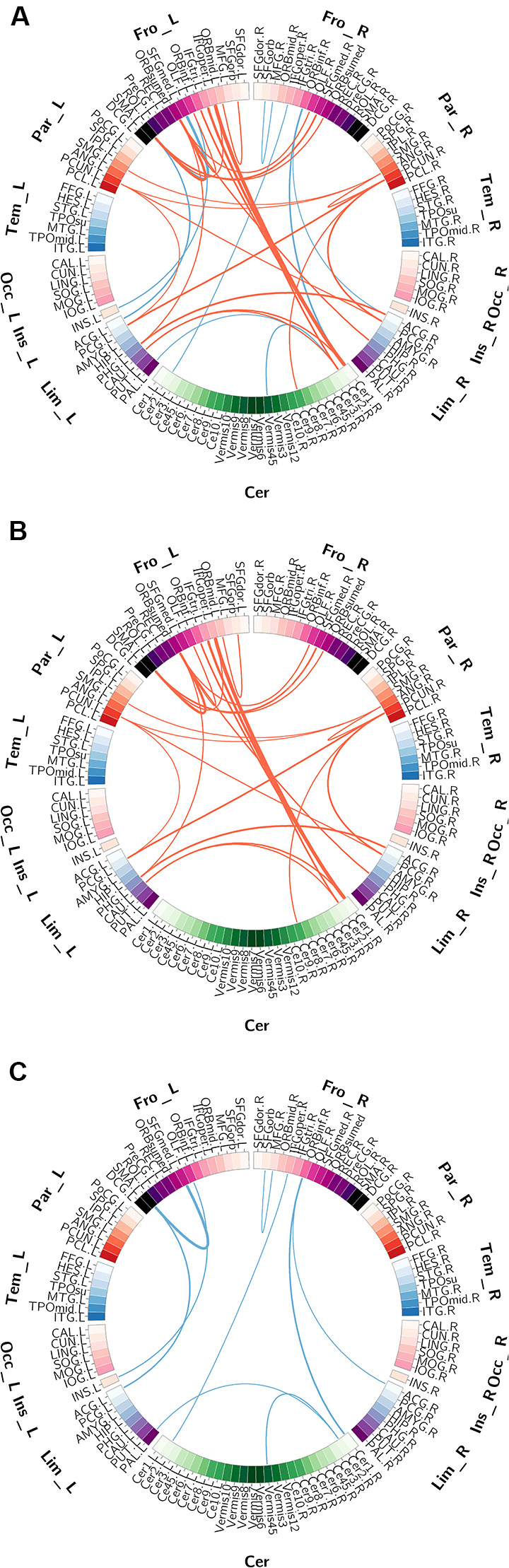
The gender differences in functional connectivity (FC) detected using two-sample *t*-tests. Panel **(A)** shows all FCs significant gender differences. Panel **(B)** shows the FCs significantly higher in females than in males. Panel **(C)** shows the FCs significantly higher in males than in females. Brain regions are represented by squares on a circle and the FCs between them are represented by the lines connecting two regions. The red lines represent higher FC in females than in males and the blue lines represent higher FC in males than in females. The thickness of the lines represents the corresponding *T*-values.

**Figure 3:**
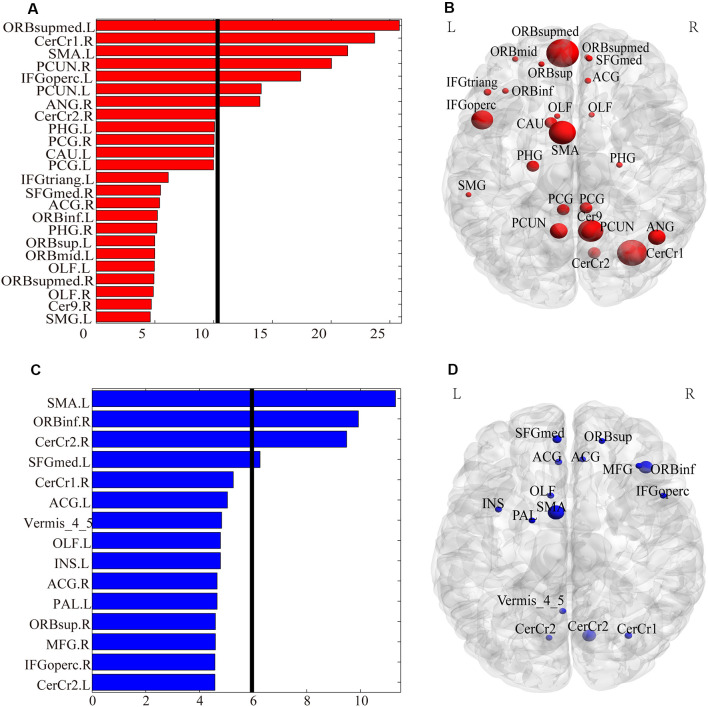
The weighted degree of the brain regions involved in the FCs with significant gender differences. Panels **(A,B)** show the bar plot and the rendering plot of the weighted degree of the regions with significantly higher values in females than in males (in red). Panels **(C,D)** show the bar plot and the rendering plot of the weighted degree of the regions with significantly higher values in males than in females (in blue). In Panels **(A,C)**, the black lines indicate the mean value. In panels **(B,D)**, the size of the spheres indicates the value of the weighted degree.

The functional connections with significant gender differences are shown in Figure 2. In total, 33 FCs showed significant differences between genders (Figure 2A), including 24 FCs showing greater strength in females than in males (Figure 2B) and 9 FCs showing greater strength in males than in females (Figure 2C). These 24 “female-stronger” FCs mainly involved frontal (13 FCs), parietal (eight FCs), limbic (nine FCs) and cerebellar (seven FCs) areas (24 areas in total). Interestingly, among the 13 “female-stronger” FCs involving frontal areas, 10 were within the left hemisphere and the other 3 were between the left and right frontal areas. The nine “male-stronger” FCs mainly involved frontal (seven FCs), limbic (three FCs), and cerebellar (four FCs) areas (15 areas in total). Among all these brain areas, the majority were frontal areas—half of the 24 areas involved in the “female-stronger” FCs and nearly two-thirds of the 15 areas involved in the “male-stronger” FCs were frontal areas. We also assessed the contribution of each brain area to the gender differences in FCs using the weighted degree. The ranked weighted degrees of these brain areas are shown in Figures 3A,C and their spatial locations are shown in Figures 3B,D. Among the 24 areas involved in the “female-stronger” FCs, seven areas, including the left medial orbital part of superior frontal gyrus, right Cerebelum_Crus 1, left Supplementary Motor Area (SMA), right precuneus, left opercular part of inferior frontal gyrus, left precuneus, right angular gyrus, had higher contributions than average (Figure 3A). Among the 15 areas involved in the “male-stronger” FCs, 4 areas, including left SMA, right orbital part of inferior frontal gyrus, right Cerebelum_Crus 2, left medial superior frontal gyrus, had higher contributions than average (Figure 3C). It should be noted that some of these areas were involved in both “female-stronger” and “male-stronger” FCs, including the left SMA, left olfactory cortex, right anterior cingulate and paracingulate gyri, right Cerebelum_Crus 1, and right Cerebelum_Crus 2.

### The Overlap of Gender Differences Between Structural and Functional Measures

**Figure 4:**
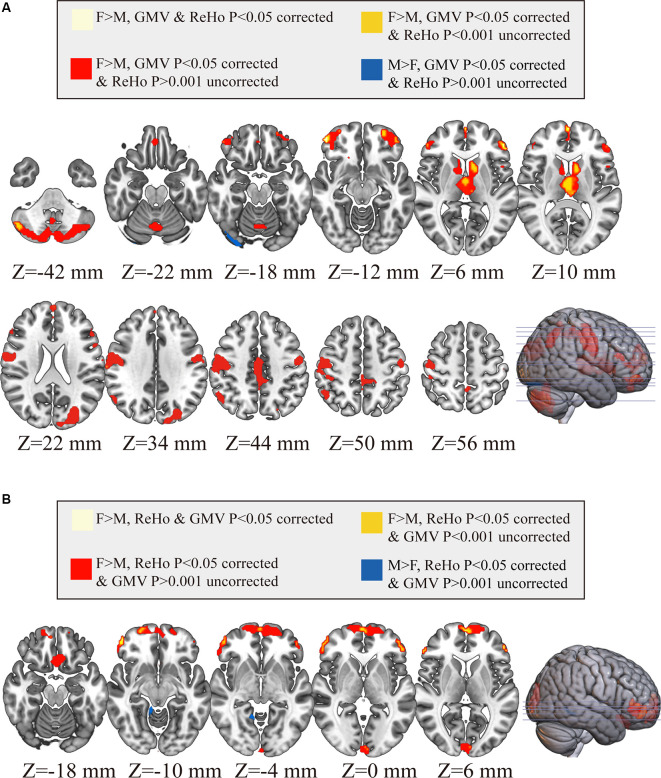
Comparisons between brain areas showing gender differences in GMV and those showing gender differences in ReHo. In Panel **(A)**, all voxels marked in color show significant gender differences in gray matter volume (GMV; *P* < 0.05 corrected; warm colors: females > males; cold color: males > females), within which the voxels in light yellow also show significant gender differences in ReHo (*P* < 0.05 correct; females > males), the voxels in orange show only a trend of gender differences in ReHo (*P* < 0.001 uncorrected; females > males) and the voxels in red (females > males for GMV) and blue (males > females for GMV) show no difference in ReHo (*P* > 0.001 uncorrected). In Panel **(B)**, all voxels marked in color show significant gender differences in ReHo (*P* < 0.05 corrected; warm colors: females > males; cold color: males > females), within which the voxels in light yellow also show significant gender differences in GMV (*P* < 0.05 correct; females > males), the voxels in orange show only a trend of gender differences in GMV (*P* < 0.001 uncorrected; females > males) and the voxels in red (females > males for ReHo) and blue (males > females for ReHo) show no difference in GMV (*P* > 0.001 uncorrected). F, female; M, males.

**Figure 5:**
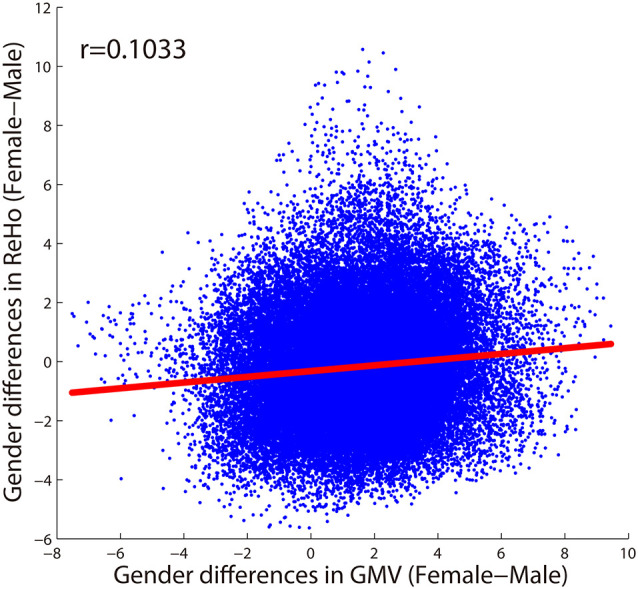
The spatial correlation between gender differences in GMV and gender differences in ReHo across voxels within the GM mask.

As shown in Figure 4, only 20 voxels were showing significant gender differences (females > males) that survived the multiple comparisons correction (*p* < 0.05, FWE corrected) in both GMV and ReHo, accounting for 0.68% of all voxels showing gender difference in GMV (light yellow in Figure 4A) and 2.75% of all voxels showing gender difference in ReHo (light yellow in Figure 4B). These 20 voxels formed three small clusters located in the right orbital part of the middle frontal gyrus, left triangular part of inferior frontal gyrus, and left medial superior frontal gyrus, respectively. When we further relaxed the statistical threshold, 16.62% of the voxels with significant GMV differences (*p* < 0.05, FWE corrected) showed a trend of gender difference in ReHo (*p* < 0.001, uncorrected; all showing increased ReHo in females than in males), which were mainly located in the thalamus and the left orbital part of middle frontal gyrus (orange in Figure 4A). When looking at the voxels showing significant ReHo differences between genders (*p* < 0.05, FWE corrected), no voxels showed a trend of gender difference in GMV after relaxing the threshold (*p* < 0.001, uncorrected). Regarding the spatial correlation analysis between gender differences in GMV and gender differences in ReHo, we observed a very weak correlation (*r* = 0.1033), shown in Figure 5.

### Gender Classification Based on GMV, ReHo, and FC

**Figure 6:**
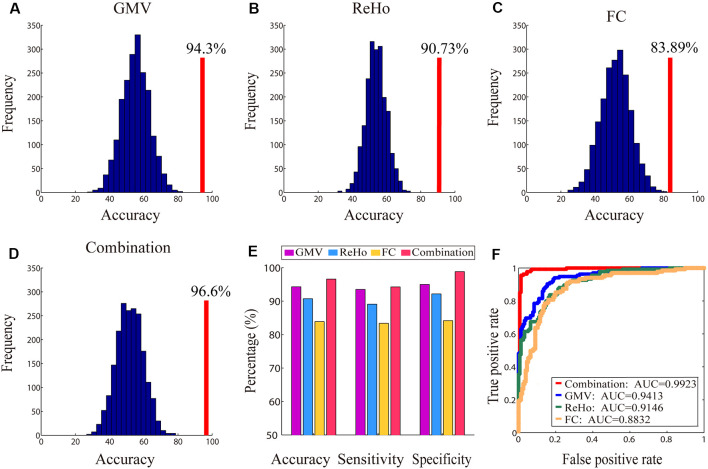
The accuracies of the classification between genders along with their corresponding null distributions for each measure (Panels **A–C**) and the combination of all measures (Panel **D**), the bar plot of all classification accuracies, sensitivity and specificity (Panel **E**) and the receiver operating characteristic (ROC) curves and corresponding area under the curves (AUCs; Panel **F**). In Panels **(A–D)**, the classification accuracies are indicated by red vertical lines and corresponding null distributions (obtained from 2,000 permutations) are indicated by blue bell-shaped distributions centered around chance level accuracy of 50%. All accuracies were statistically significant (*p* < 0.001).

The classification results obtained based on single, as well as combined, brain imaging metrics are shown in Figure 6. The classification using the GMV, ReHo, and FC separately yielded CAs of 94.3%, 90.73%, and 83.89%, respectively (indicated by red vertical lines in Figures 6A–C). These CAs were obtained when 13,200 voxels (GMV), 11,400 voxels (ReHo), and 720 FCs were selected, respectively. The corresponding AUCs for the three classifications were 0.9413 (GMV), 0.9146 (ReHo) and 0.8832 (FC), respectively (Figure 6F). The classification using the combination of the three imaging measures yielded a higher CA of 96.6% (indicated by the red vertical line in Figure 6D; when 8,000 features were selected) and a higher AUC of 0.9923 (Figure 6F). All these CAs were significantly higher than chance level (*P* < 1/2,000 = 0.0005, 2,000 permutation tests; the null distributions are indicated by the blue bell-shape in Figures 6A–D and corrected using Bonferroni correction).

Combining different imaging metrics also improves both sensitivity and specificity (Figure 6E)—the sensitivities obtained from GMV, ReHo, FC and their combination were 93.5%, 89.1%, 83.38% and 94.27%, respectively, and the specificities obtained from GMV, ReHo, FC and their combination were 95%, 92.17%, 84.17% and 98.83%, respectively.

## Discussion

In the present study, we examined the gender differences in both brain structural and functional measures with a focus on the similarities and differences between them. We obtained three main findings. First, using the same group of participants, gender differences were detected in both structural (i.e., GMV) and functional (ReHo or FC) imaging measures, mainly manifested as greater values in females than in males in regions of the frontal, parietal, occipital lobes and cerebellum. Second, there was essentially very little overlap of gender differences between structural and functional measures, indicating that gender differences were manifested differently in brain structure and function. Finally, successful gender classification was obtained using each of the brain structural and functional measures; moreover, their combination could further improve the classification performance to some extent, suggesting that different brain imaging measures might contain complementary information about gender differences.

### Structural and Functional Differences Between Genders

We observed gender differences in both structural and functional imaging measures. We found that gender-differences in structural and functional measures were mainly manifested as greater values in females than in males (i.e., greater GMV or ReHo values, or stronger FCs in females). This observation is consistent with many previous studies using subjects including young adults as in the present study (Good et al., 2001; Sowell et al., 2007; Lv et al., 2010; Im et al., 2006; Luders et al., 2006; Feis et al., 2013). It is worth noting that this gender difference (i.e., predominantly manifested as stronger values in females than in males) was not observed in a recent study with a very large sample (*n* = 5216). However, the subjects used in this previous study were much older (44–77 years) than the present study (18–29 years), suggesting that the effect of greater structural and functional measures in females than in males may be age-related (Kawachi et al., 2002; Sowell et al., 2007; Zuo et al., 2010; Gennatas et al., 2017).

Here, the gender differences in brain structure and function detected in the present study were mostly located in the frontal regions—stronger in females than in males. Greater values of structural and/or functional measures in frontal areas in females have been reported by many previous studies. For example, a meta-analysis of sex differences in brain structure reported that females on average had larger GMV in the right inferior frontal pole, middle frontal gyrus and orbitofrontal cortex (Ruigrok et al., 2014). Moreover, two studies found increased cortical thickness in prefrontal gyrus in females compared with males (Luders et al., 2006; Lv et al., 2010). Regarding functional measures, our result was also in concert with a PET study showing stronger glucose metabolism of the frontal lobe in women than in men (Andreason et al., 1994). There was also a study that examined gender difference in ReHo and found some prefrontal areas showing greater ReHo in females as in the present study; however, they also reported some other prefrontal areas showing greater ReHo in males than in females, which was not observed in the present study (Wang et al., 2012). The frontal regions were pertinent to many higher cognitive functions. Especially, the orbitofrontal area has been demonstrated to play a critical role in emotion processing and decision making (Bechara et al., 2000). In the present study, we found that females had greater GMV and ReHo in the orbitofrontal area than males, which may be responsible for higher emotion perception ability in females than in males (Stevens and Hamann, 2012).

Besides, consistent with several previous findings, we also found that sensorimotor-related regions showed larger GMV in females, including the thalamus (Feis et al., 2013; Ruigrok et al., 2014), postcentral gyrus (Lv et al., 2010), inferior parietal lobule (Good et al., 2001; Chen et al., 2007), mid-cingulate gyrus (Feis et al., 2013), caudate (Good et al., 2001; Luders et al., 2009; Wang et al., 2012; Feis et al., 2013) and cerebellum (Wang et al., 2012). It should be noted that inconsistent results also exist, especially for the cerebellum which was reported to have smaller GMV in females (Giedd et al., 2012). Among these areas, the postcentral gyrus, inferior parietal lobule, and the mid-cingulate gyrus were also reported to have greater ReHo in females in a previous study (Xu et al., 2015); however, these regions did not show gender difference in ReHo in the present study. In terms of FC, interestingly, the SMA and cerebellum were found to be associated with both “female-stronger” FCs and “male-stronger” FCs. These FCs showing gender differences were mainly between SMA/cerebellum and different prefrontal areas, that is, some prefrontal areas showed stronger connectivity with SMA/cerebellum in females and some other prefrontal areas showed stronger connectivity with SMA/cerebellum in males. In general, the prefrontal cortex serves a critical role in the coordination and execution of motor actions *via* its involvement in goal setting, decision-making, motivation, and cognitive control, and its connectivity with SMA/cerebellum may be related to the integration between higher cognitive action control and motor performance (Grafton and Volz, 2019). However, it has been suggested that different part of the prefrontal cortex may be involved differently in females and males during certain cognitive-motor control tasks (Rubia et al., 2013; Koch et al., 2007), which may explain the present finding that, for the FCs between prefrontal areas and SMA/cerebellum, some were stronger in females and some were stronger in males.

Regarding the gender differences in FCs detected in the present study, it is worth noting that females exhibited stronger FCs related to the DMN (seven FCs were related to precuneus and posterior cingulate cortex; Figure 2B), which was also reported in several previous studies (Xu et al., 2015; Ritchie et al., 2018). The DMN has been widely recognized to be correlated with some cognitive functions, such as social cognition and episodic memory (Kennedy and Adolphs, 2012; Sestieri et al., 2011). It has been suggested that females had advantages in these cognitive domains relative to males (Gur et al., 2012), which might be explained by the stronger DMN-related FCs found in females.

Furthermore, we also observed some lateralization of gender differences in brain structural and functional measures. More specifically, we observed lateralization of gender differences in the occipital lobe for both GMV and ReHo—females showed larger GMV and ReHo only in the left occipital areas and males showed larger GMV and ReHo only in the right occipital areas. Also, the frontal areas associated with the “female-stronger” FCs were mostly within the left hemisphere (Figure 3B). This observed lateralization was compatible with a previous study on gender differences in functional connectivity density (FCD) showing that females had greater leftward lateralization of FCD in the inferior frontal cortex, whereas males had greater rightward lateralization in inferior frontal, superior temporal and inferior occipital cortices (Tomasi and Volkow, 2012). It has been demonstrated that visual-spatial function was featured by right-hemisphere lateralization and greater rightward lateralization was associated with better performance in visual-spatial tasks (Gur et al., 1994; Wendt and Risberg, 1994), and the language-related function was featured by the left inferior frontal cortex (Hjelmervik et al., 2012). Interestingly, males generally perform better than females on visual-spatial tasks (Linn and Petersen, 1985; Voyer et al., 1995), whereas females generally outperform males in verbal tasks (Hyde and Linn, 1988; Hjelmervik et al., 2012). This gender superiority in spatial and language functions may be accounted for by the lateralization of brain structure and function between genders observed in the present and previous studies.

All these observed gender differences in brain structure and function may be related to behavioral differences between genders. Using the same dataset, our previous studies have found significant differences between genders in five domains of personality traits: Sensation Seeking Scale (SSS), Eysenck Personality Questionnaire (EPQ), Emotional Intelligence Scale (EIS), Beck Depression Inventory (BDI) and Tridimensional Personality Questionnaire (TPQ). Specifically, males showed higher scores in three subscales of the SSS including the adventure-seeking subscale (Zhou et al., 2014), experience seeking subscale (Zhou et al., 2014) and disinhibition subscale (Zhou et al., 2014), one subscale of the EPQ that is the extraversion-introversion subscale (Zhou et al., 2014), the EIS (Wu et al., 2016) and BDI (Wu et al., 2016); females showed higher scores in the neuroticism subscale of the EPQ (Zhou et al., 2014) and the harm avoidance subscale of the TPQ (Li et al., 2012). Furthermore, the relationships between brain image measures and behavioral data related to gender effects have also been identified. For example, an interaction between gender and risk propensity was found for short- and long-range FC densities in the left inferior orbitofrontal cortex and right supramarginal gyrus/postcentral gyrus, implying that the resting-state neural correlates of risk propensity may differ between men and women (Zhou et al., 2014). Also, gender differences were found in the correlations between the harm avoidance score and the resting-state FCs of the amygdala (Li et al., 2012) and in the correlations between the emotion regulation and the FCs associated with the amygdala (Wu et al., 2016).

### Little Overlap of Gender Differences Between Structural and Functional Measures

Although females and males showed differences in the above brain areas for structural (i.e., GMV) and/or functional (i.e., ReHo and FC) measures, surprisingly, there was very little overlap between the brain areas showing structural (i.e., GMV) differences and those showing functional (i.e., ReHo and FC) differences between genders (Figures 3, 4). Indeed, only 20 voxels showed significant gender differences in both GMV and ReHo when they were properly thresholded (i.e., corrected for multiple comparisons), accounting for a very small percentage of all voxels showing gender differences in GMV (0.68%) or ReHo (2.75%). Even when the statistical threshold was relaxed, still only a small percentage (16.62% or 0%) of the voxels showing significant differences in GMV or ReHo showed a trend of differences in the other measure. This was further corroborated by the observation of a very weak spatial correlation (*r* = 0.1033) between gender differences in GMV and gender differences in ReHo.

These results suggest that, although some brain areas were structurally different between females and males, they did not exhibit a significant difference in functional measures, and *vice versa*. This is somewhat unexpected as it is usually considered that brain structure and function are closely related (Castagna, 2019). However, dissociations between structural measures and functional measures are also often reported (Owens et al., 2018). One possible explanation is that the development of the structure and function of the human brain may be affected by environment and personal experience to different degrees (Mitchell et al., 2011; Koziol et al., 2014; Sale et al., 2014). Consequently, gender differences in ReHo cannot be fully accounted for by gender differences in GM (Wang et al., 2012). This further raises the possibility that brain structural and functional measures contain complementary information about gender that may be utilized for a better prediction of a person’s gender.

### Combining Different Brain Imaging Measures Better Predicts the Gender

Recently, the application of MVPA strategy based on machine learning techniques in analyzing neuroimaging data has attracted a lot of attention. Relying on the spatial patterns of multiple variables of interest, the MVPA strategy has been proven to have higher sensitivity in information detection than univariate approaches which only evaluate a single variable at a time, mainly for two reasons. First, the spatial pattern composed of multiple variables contains information from multiple dimensions (i.e., variables) and thus contains more information than every single variable. Second, MVPA analyses multiple variables at once and thus is not exposed to multiple comparisons problem. This multivariate nature of MVPA makes it a natural way to combine information from different sources. The results of the univariate comparisons of brain structural and functional measures between genders in the present study suggest that gender differences in brain structure and brain function may provide different and complementary information.

In the present study, we observed that the gender classification accuracies based on each type of brain imaging measures were 94.3% for GMV, 90.73% for ReHo and 83.89% for FC, and all these accuracies were significantly higher than chance level. This indicates that each of these brain structural and functional measures contains gender-distinct information. More importantly, the CA was further improved to 96.6% when combining all three measures which were higher than the highest CA obtained from a single measure (i.e., 94.3% for GMV). This improvement suggests a higher predictive power might be achieved by merging structural and functional information provided by different imaging measures, which needs to be confirmed by future studies.

## Conclusion

Our results revealed that, although females and males showed differences in both brain structure and function in widely distributed brain areas, such gender differences in brain structure and those in brain function were very different. The observation of a better classification performance obtained by combining these different brain imaging measures further confirmed that structural and functional measures contained complementary information about gender differences. These results highlight the complex relationship between brain structure and function, which may underlie the complex nature of gender differences in behavior.

## Data Availability Statement

The datasets generated for this study are available on request to the corresponding author.

## Ethics Statement

The studies involving human participants were reviewed and approved by the Medical Research Ethics Committee of Tianjin Medical University General Hospital. The participants provided their written informed consent to participate in this study.

## Author Contributions

XZ and ML: study design and article writing. WQ: technical guidance. BW and CY: supervision. ML and DM: manuscript review and editing. All authors approved the final version of the article to be published.

## Conflict of Interest

The authors declare that the research was conducted in the absence of any commercial or financial relationships that could be construed as a potential conflict of interest.

## Footnotes

^1^http://dbm.neuro.uni-jena.de/vbm8/

^2^http://www.fil.ion.ucl.ac.uk/spm

^3^http://rfmri.org/DPARSFA
